# Supplementary material for: From sequence to enzyme mechanism using multi-label machine learning
Source: BMC Bioinformatics. 2014 May 19;15:150. doi: 10.1186/1471-2105-15-150 (PMC4229970; doi:10.1186/1471-2105-15-150)
Supplement: Additional file 2 — Java code of ml2db. Additional file ml2db_code.tar.gz contains the Java source code to run the multi-label machine learning experiments and save the results to database. The code’s Javadoc is included. [file 1471-2105-15-150-S2.zip › additional file 2/ml2db/utils/doc/uk/ac/standrews/utils/main/database/TableManager.html]

TableManager


JavaScript is disabled on your browser.


- Overview
- Package
- Class
- Use
- Tree
- Deprecated
- Index
- Help

- Prev Class
- Next Class

- Frames
- No Frames

- All Classes

- Summary:
- Nested |
- Field |
- Constr |
- Method

- Detail:
- Field |
- Constr |
- Method


uk.ac.standrews.utils.main.database

## Class TableManager

- java.lang.Object
- - uk.ac.standrews.utils.main.database.Manager
  - - uk.ac.standrews.utils.main.database.DbManager
    - - uk.ac.standrews.utils.main.database.TableManager

- All Implemented Interfaces:
  :   java.io.Serializable

  Direct Known Subclasses:
  :   TableCreator, TableReader, TableRow, TableWriter

  ---

    

  ```
  public class TableManager
  extends DbManager
  ```

  Class to create/read/write to a database table.

  Version:
  :   10 Jun 2010

  Author:
  :   Luna De Ferrari luna.deferrari-at-ed.ac.uk

  See Also:
  :   Serialized Form

- - ### Field Summary

    - ### Fields inherited from class uk.ac.standrews.utils.main.database.DbManager

      `m_dbWriter`
  - ### Constructor Summary

    Constructors

    Constructor and Description

    `TableManager(Table table)`

    Constructor to create a manager to create, read and write a table.
  - ### Method Summary

    Methods

    | Modifier and Type | Method and Description |
    | --- | --- |
    | `Table` | `getTable()` |
    | `java.lang.String` | `getTableName()` |

    - ### Methods inherited from class uk.ac.standrews.utils.main.database.DbManager

      `closeConnection, connectionIsValid, getDatabaseName, getDbConnection, getDbCreator, getDbReader, getDbWriter, refreshConnection, runAnalyzeTable`
    - ### Methods inherited from class java.lang.Object

      `equals, getClass, hashCode, notify, notifyAll, toString, wait, wait, wait`

- - ### Constructor Detail


    - #### TableManager

      ```
      public TableManager(Table table)
      ```

      Constructor to create a manager to create, read and write a table.

      Parameters:
      :   `dbManager` -
      :   `table` -
  - ### Method Detail


    - #### getTableName

      ```
      public java.lang.String getTableName()
      ```


    - #### getTable

      ```
      public Table getTable()
      ```


- Overview
- Package
- Class
- Use
- Tree
- Deprecated
- Index
- Help

- Prev Class
- Next Class

- Frames
- No Frames

- All Classes

- Summary:
- Nested |
- Field |
- Constr |
- Method

- Detail:
- Field |
- Constr |
- Method
